# NAD^+^ Metabolism as an Emerging Therapeutic Target for Cardiovascular Diseases Associated With Sudden Cardiac Death

**DOI:** 10.3389/fphys.2020.00901

**Published:** 2020-08-13

**Authors:** Weiyi Xu, Le Li, Lilei Zhang

**Affiliations:** ^1^Department of Molecular and Human Genetics, Baylor College of Medicine, Houston, TX, United States; ^2^Department of Anesthesiology, Zhujiang Hospital, Southern Medical University, Guangzhou, China

**Keywords:** NAD^+^, NAMPT, sirtuin, myocardium, sudden cardiac death, ischemia reperfusion, heart failure, arrhythmia

## Abstract

In addition to its central role in mediating oxidation reduction in fuel metabolism and bioenergetics, nicotinamide adenine dinucleotide (NAD^+^) has emerged as a vital co-substrate for a number of proteins involved in diverse cellular processes, including sirtuins, poly(ADP-ribose) polymerases and cyclic ADP-ribose synthetases. The connection with aging and age-associated diseases has led to a new wave of research in the cardiovascular field. Here, we review the basics of NAD^+^ homeostasis, the molecular physiology and new advances in ischemic-reperfusion injury, heart failure, and arrhythmias, all of which are associated with increased risks for sudden cardiac death. Finally, we summarize the progress of NAD^+^-boosting therapy in human cardiovascular diseases and the challenges for future studies.

## Introduction

Sudden cardiac death (SCD) is defined as a sudden, unexpected death caused by loss of cardiac function (Zipes and Wellens, 1998; Zipes et al., 2006). SCD is responsible for 300,000 – 400,000 deaths each year in the United States with an annual incidence of 60 per 100,000 (Stecker et al., 2014). Most SCD events occur in patients who were not previously identified as being at risk for SCD (Piccini Sr et al., 2016). Despite the improving resuscitation rate and the increasing use of implantable cardioverter defibrillator, the majority who suffer a SCD will not survive. SCD remains a major international public health problem in clinical cardiology, emergency medicine, and public health (Lown, 1979; Moss, 1980).

Nicotinamide adenine dinucleotide or NAD^+^, is one of the most essential small molecules in mammalian cells. NAD^+^ interacts with over 500 enzymes (Ansari and Raghava, 2010) and plays important roles in almost every vital aspect in cell biology and human physiology (Katsyuba et al., 2020). Dysregulation of NAD^+^ homeostasis is associated with a number of diseases including cardiovascular diseases (CVD) (Hershberger et al., 2017; Matasic et al., 2018; Rajman et al., 2018; Katsyuba et al., 2020; Ralto et al., 2020). Particularly, modulation of NAD^+^ metabolism has been proposed to provide beneficial effects for CVD settings that are highly associated with SCD, such as ischemia/reperfusion injury (I/R injury), heart failure and arrhythmia (Hershberger et al., 2017; Matasic et al., 2018). In this review, we will discuss how alteration in NAD^+^ metabolism can lead to heart disease with the focus on I/R injury, heart failure, and arrhythmia. We will also provide a comprehensive review on animal and human studies with NAD^+^ boosters, and discuss the feasibility of NAD^+^-boosting therapy for SCD-associated CVD.

## NAD^+^ Metabolism in the Heart

### NAD^+^ Biosynthesis

**FIGURE 1:**
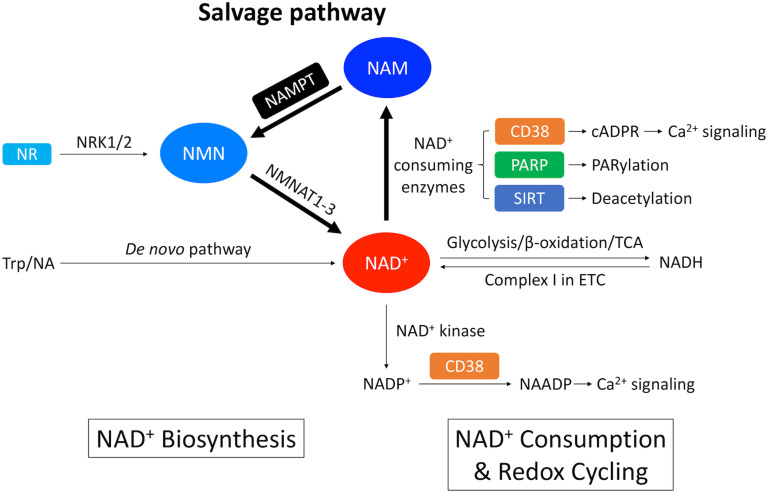
Overview of NAD^+^ metabolism in the heart. NAD^+^ is synthesized through the *de novo* pathway and the salvage pathway in mammalian cells. The *de novo* pathway generates NAD^+^ from Trp or NA. The salvage pathway, indicated by the thicker arrows, is the major NAD^+^ biosynthesis pathway in the heart where NAM generated from NAD^+^-consuming enzymes is converted into NMN through NAMPT – the rate limiting enzyme. NMN can also be generated from NR by NRK1/2. NMN is further converted into NAD^+^ by NMNAT1-3. A redox potential between NAD^+^ and its reduced form NADH is established by key catabolic processes including glycolysis, fatty acid β-oxidation and TCA cycle, which drives the electron transport chain (ETC). On the other hand, NAD^+^ is consumed by NAD^+^-consuming enzymes, including CD38 that generates cADPR, which is a Ca^2+^-mobilizing second messenger, PARP that catalyzes protein PARylation and SIRT that catalyzes protein deacetylation. These enzymatic reactions produce NAM as a byproduct that can be recycled back into NAD^+^ salvage pathway. In addition, NAD^+^ can also be consumed by NAD^+^ kinase which generates NADP^+^. Through CD38, NADP^+^ is further converted into NAADP, which is another Ca^2+^-mobilizing second messenger.

The heart, along with the kidney and the liver has the highest level of NAD^+^ among all the organs (Mori et al., 2014). In mammalian cells, NAD^+^ is synthesized via two distinct pathways: the *de novo* pathway and the salvage pathway (Figure 1; Sporty et al., 2009). The *de novo* pathway generates NAD^+^ from tryptophan (Trp) through the kynurenine metabolic pathway (Badawy, 2017), or nicotinic acid (NA) through the Preiss-Handler pathway (Preiss and Handler, 1958a, b). Nevertheless, most of the extrahepatic organs including the heart, use the salvage pathway as the main route to generate NAD^+^ (Mori et al., 2014; Liu et al., 2018). Mori et al. (2014) established the metabolic profiling of NAD^+^ biosynthetic routes in mouse tissues by measuring the *in vitro* activity of enzymes, the levels of substrates and products, and revealed that 99.3% of NAD^+^ in the heart is generated by the salvage pathway. On the other hand, enzymes involved in the *de novo* pathway are of low expression and low activity in the heart (Ikeda et al., 1965).

**FIGURE 2:**
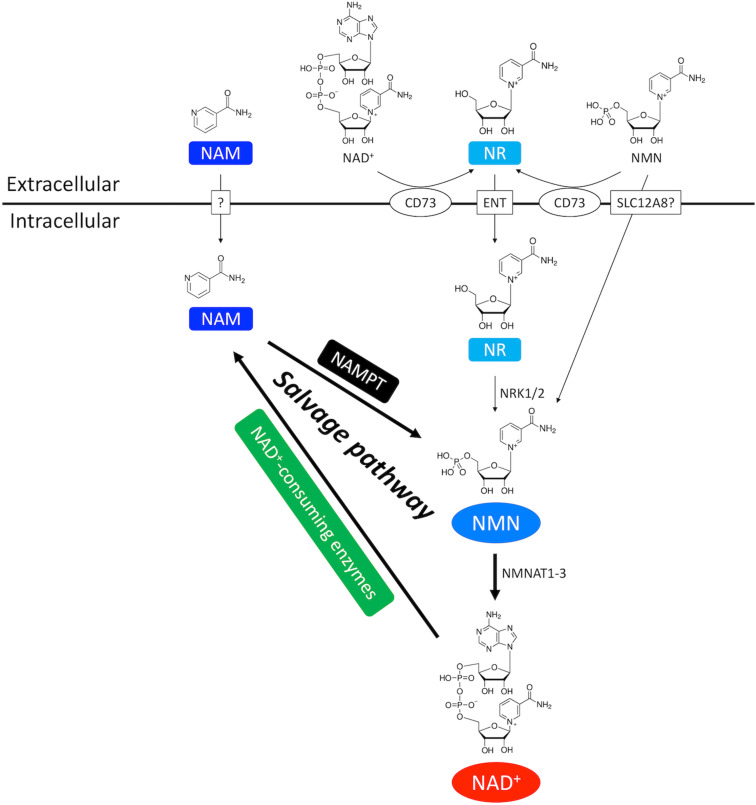
NAD^+^ salvage pathway in the heart. NAM and NMN are two important intermediate NAD^+^ metabolites in the NAD^+^ salvage pathway. NAM is the byproduct of NAD^+^ consumption. Exogenous NAM can also enter the cytoplasm through a putative membrane transporter. NMN comes from three routes. First, NMN can be generated from NAM by the rate-limiting enzyme NAMPT. Second, extracellular NMN can be transported into cytoplasm through a membrane transporter which might be SLC12A8. Third, NMN can be generated from NR by NRK1/2. Extracellular NR, either from direct supplementation or CD73-mediated conversion from extracellular NMN and NAD^+^, is transported into cells through equilibrative nucleoside transporter (ENT).

Salvage pathway generates NAD^+^ from the NAD^+^ degradation product nicotinamide (NAM) (Figures 1, 2; Ralto et al., 2020). NAM is converted into an intermediate product nicotinamide mononucleotide (NMN) via NAM phosphoribosyltransferase (NAMPT) – the rate limiting enzyme in the salvage pathway. Extracellular NMN can also be transported into the cells through a NMN transporter which might be solute carrier family 12 member 8 (SLC12A8) (Grozio et al., 2019). NMN is then converted into NAD^+^ by NMN adenyltransferase 1-3 (NMNAT1-3) in the final step. In addition, NMN can also be generated from another NAD^+^ precursor nicotinamide riboside (NR) by NR kinase 1/2 (NRK1/2) (Figures 1, 2). Cardiac expression of NRK2 is much higher than NRK1 (Ratajczak et al., 2016), suggesting that NRK2 may control the phosphorylation of NR in the heart. Moreover, NR can be generated from extracellular NAD^+^ or NMN through CD73 (Grozio et al., 2013). Both NMN and NR preserve the pyridine ring, and thus do not require NAMPT to be converted back to NAD^+^. Details of the NAD^+^ salvage pathway is illustrated in Figure 2.

### The Rate-Limiting Enzyme for the Salvage Pathway: NAMPT

Much attention has been drawn to NAMPT as it directly correlates with the NAD^+^ levels in the heart (Revollo et al., 2004). For instance, *Nampt* expression and NAD^+^ level are both reduced in the heart after I/R injury, and ectopic expression of *Nampt* restored the NAD^+^ level (Hsu et al., 2009).

**FIGURE 3:**
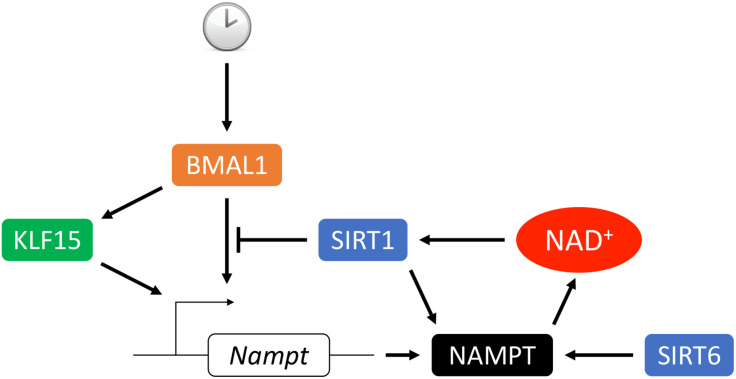
Circadian transcriptional regulation of NAMPT. The circadian rhythmic transcription of *Nampt* in the heart depends on intricate regulation from the core clock. BMAL1 complex binds to the promoter E-box and activates *Nampt* transcription which leads to upregulation of NAMPT expression, NAD^+^ biosynthesis and SIRT1 activity. In turn, SIRT1 deacetylates BMAL1 and histone H3 at the *cis*-acting site which suppresses the *Nampt* transcription. Transcription factor KLF15 integrates inputs from both the core clock and additional metabolic cues to promote *Nampt* transcription via direct binding of the enhancer region of *Nampt* in intron 1. Additionally, activity and secretion of NAMPT can be stimulated by SIRT1- or SIRT6-mediated deacetylation.

NAMPT expression oscillates in a circadian fashion in the heart (Um et al., 2011; Young et al., 2014; Peliciari-Garcia et al., 2016; Li et al., 2020). Previous studies have shown that *Nampt* gene expression is under the regulation of core clock machineries, circadian locomoter output cycles protein kaput (CLOCK) and brain and muscle Arnt-like protein 1 (BMAL1), as well as SIRT1, which leads to rhythmic cellular NAD^+^ levels (Nakahata et al., 2009; Ramsey et al., 2009; Wijnen, 2009). CLOCK:BMAL1 transcription complex binds to E-box on the *Nampt* promoter and activates its expression. Upregulation of NAMPT increases NAD^+^ production, which as a co-factor activates SIRT1 activity. In turn, SIRT1 deacetylates BMAL1 and histone H3 at the cis-acting site and silences the *Nampt* gene expression. Thus, NAD^+^ connects the two feedback loops of circadian rhythmic gene expression and cellular metabolism (Figure 3). Furthermore, our recent study showed that Kruppel-like factor 15 (KLF15), a zinc finger transcription factor also directly regulates *Nampt* at the transcriptional level in a circadian fashion by binding to an enhancer element of *Nampt* in intron 1 (Li et al., 2020). It is interesting that KLF15 deficiency only abolishes the circadian rhythmic expression of *Nampt* and does not affect its baseline expression, while in BMAL1 knockout mice overall expression of *Nampt* is reduced (Ramsey et al., 2009). This may be explained by the observation that KLF15 and BMAL1 each binds to a different cis-regulatory element (Figure 3). And while KLF15 binding may be spared for basal level of *Nampt* expression during the resting phase, it is required for optimal *Nampt* expression during the active phase, when the metabolic demand is high. In addition, the oscillatory expression of *Nampt* is absent in AMPKα2 but not in AMPKα1 knockout mice, suggesting that AMPKα2 also participates in the regulation of circadian *Nampt* expression in the heart (Um et al., 2011) possibly through the core clock suppressors cryptochrome 1/2 (Lamia et al., 2009) and period 2 (Um et al., 2007).

Another intriguing feature about NAMPT is that it can also be secreted by multiple cell types, including the adipocytes (Revollo et al., 2007), hepatocytes (Garten et al., 2010), monocytes (Schilling and Hauschildt, 2012) and pancreatic β cells (Kover et al., 2013), and be present as an extracellular form (eNAMPT) in circulation. eNAMPT is secreted in extracellular vesicles (Yoshida et al., 2019), which may then be internalized by the recipient cells (Lu et al., 2019), thus elevates NAD^+^ biosynthesis in adjacent tissues through paracrine effects (Lu et al., 2019; Yoshida et al., 2019), or even in remote tissues through circulation (Revollo et al., 2007; Imai, 2016; Yoshino et al., 2018). Secretion of eNAMPT can be enhanced by SIRT1- (Yoon et al., 2015) or SIRT6 (Sociali et al., 2019)-mediated deacetylation, which also increases the NAMPT enzymatic activity. On the other hand, eNAMPT has also been reported as a proinflammatory cytokine through mechanisms that are independent of its NAD^+^ biosynthetic activity (Grolla et al., 2016; Carbone et al., 2017; Dalamaga et al., 2018; Yoshino et al., 2018). In the heart, cardiomyocytes (Pillai et al., 2013; Hsu et al., 2014), perivascular (Wang et al., 2009) and epicardial adipose tissues (Cheng et al., 2008) were shown to secrete eNAMPT. Whether eNAMPT is beneficial or detrimental to heart function remains controversial (Montecucco et al., 2013; Pillai et al., 2013; Yano et al., 2015).

### NAD(P)^+^-NAD(P)H Redox Cycling

**FIGURE 4:**
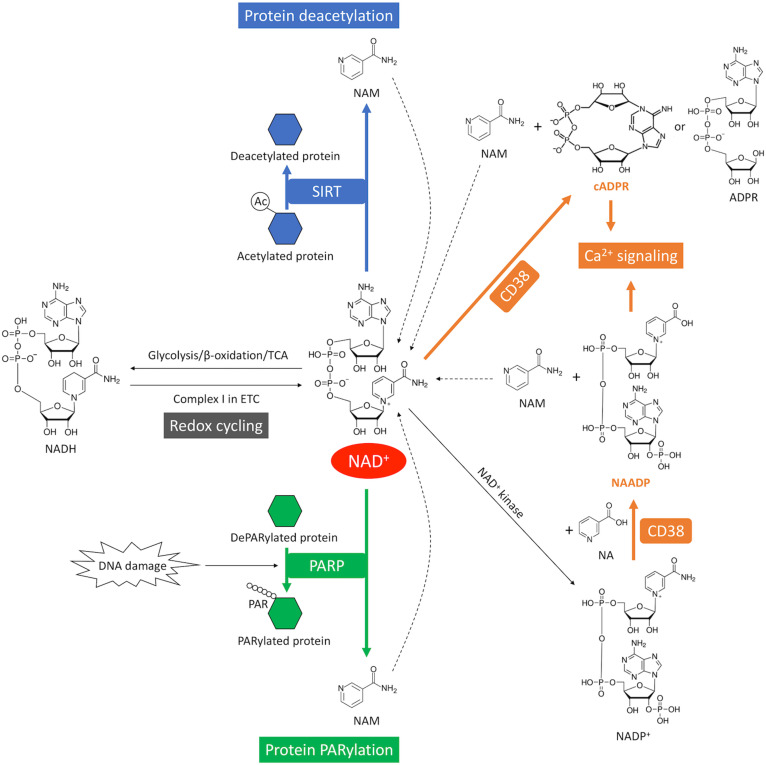
NAD^+^ consumption and redox cycling in the heart. SIRT mediates NAD^+^ consumption during deacetylation, where the acetyl group is removed from the acetylated protein (blue). DNA damage activates PARP-mediated NAD^+^ consumption during PARylation, where the ADP-ribose chain is added onto the target proteins (green). CD38-mediates NAD^+^ consumption through cyclization or base-exchange reaction, where two important Ca^2+^-mobilizing second messengers, cADPR or NAADP, are produced from NAD^+^, respectively (orange). CD38 also generates ADPR from NAD^+^ through its glycohydrolase activity. NAM is generated as a byproduct from NAD^+^ consumption. The dash lines indicate recycling of NAM back into NAD^+^ salvage pathway. The redox cycling between NAD^+^ and NADH is indicated in gray.

NAD^+^ is a hydride acceptor that can be transformed into its reduced form NADH after accepting an electron (Figures 1, 4). Reduction of NAD^+^ to NADH occurs during fuel catabolism. Fatty acid is the primary fuel source in the heart under physiological conditions (Grynberg and Demaison, 1996). β-oxidation of fatty acid and tricarboxylic acid (TCA) cycle reduce NAD^+^ to NADH. NADH is then fed into the electron transport chain to generate ATP in the mitochondria while being oxidized back to NAD^+^. NAD^+^ depletion reduces ATP content in cardiomyocytes (Hsu et al., 2009). Severe NAD^+^ depletion (>95%) may even disable the ability of ATP generation in cells (Del Nagro et al., 2014).

In addition, NAD^+^ can be phosphorylated by NAD^+^ kinase and converted into nicotinamide adenine dinucleotide phosphate (NADP^+^) (Figures 1, 4). A redox cycling is also established between NADP^+^ and NADPH. NADP^+^ is mostly consumed in the pentose phosphate pathway (PPP) where it is reduced into NADPH. However, the capacity of PPP is very small in the heart due to the low activity of the rate-limiting enzyme glucose-6-phosphate dehydrogenase (Zimmer, 1992). NADPH is the substrate of NADPH oxidases (NOXs). NOXs are the major reactive oxygen species (ROS)-generating enzymes in the heart and have emerged as the primary source of oxidative stress underlying a variety of CVD (Zhang et al., 2020).

### NAD^+^ Consumption and NAD^+^-Consuming Enzymes

The homeostasis of NAD^+^ relies on the balance between NAD^+^ biosynthesis and NAD^+^ consumption. There are three major types of enzymes that utilize NAD^+^ as a co-substrate, including cyclic ADP-ribose synthetases which is mainly CD38 in the heart (Lin et al., 2017), poly ADP-ribose (PAR) polymerases (PARPs) and sirtuins (SIRTs) (Figures 1, 4). These enzymatic reactions convert NAD^+^ to NAM which must be recycled through salvage pathway by rate-limiting enzyme NAMPT (Figures 1, 4). It is important to note that different NAD^+^-consuming enzymes have different enzymatic kinetics or Michaelis constant (K_m_) for NAD^+^. Therefore, whether activation of a particular enzyme can actually lead to rapid depletion of NAD^+^ would depend on the quantitative relationship between the K_m_ value of that particular enzyme and the physiological level of NAD^+^ as detailed below (Katsyuba et al., 2020).

### CD38 and Ca^2+^ Signaling

CD38 is a multifunctional enzyme (Wei et al., 2014; Lin et al., 2017; Hogan et al., 2019) that metabolizes NAD^+^ to ADP-ribose (ADPR) through its glycohydrolase activity (Kim et al., 1993; Takasawa et al., 1993) or cyclic ADP-ribose (cADPR) through its cyclase activity (Howard et al., 1993). In the presence of NA, CD38 can also mediate a base-exchange reaction in which NADP^+^ is converted to nicotinic acid adenine dinucleotide phosphate (NAADP) (Aarhus et al., 1995). The major activity of CD38 is the hydrolysis of NAD^+^ into ADPR (Dousa et al., 1996; Graeff et al., 2009). The K_m_ value of CD38 [16 μM (Sauve et al., 1998), 26 μM (Cakir-Kiefer et al., 2001)] for NAD^+^ is far below physiological range of intracellular NAD^+^ level (400–700 μM) (Hara et al., 2019), thus activation of CD38 can rapidly consume and deplete NAD^+^ content. Aging and inflammation are associated with upregulated CD38 gene expression and declined NAD^+^ level (Chini et al., 2018).

In the heart, CD38 expression is highest in the endothelial cells, with a relative enzymatic activity ratio of 100:20:1 in endothelium, cardiac fibroblasts and cardiomyocytes, respectively (Boslett et al., 2018). Activation of CD38 by hypoxia-reoxygenation depletes NAD(P)^+^ in cardiac endothelial cells and impairs nitric oxide production by endothelial nitric oxide synthase that utilizes NADPH as the reducing substrate (Forstermann and Sessa, 2012), suggesting that CD38 may cause endothelial dysfunction after ischemic injury (Boslett et al., 2018). In contrast, a CD38 inhibitor luteolinidin preserved NAD(P)^+^ level, endothelial and myocardial function in an *ex vivo* heart I/R model (Boslett et al., 2017). The cardioprotective effect of CD38 deficiency have been shown to be SIRT1- (Guan et al., 2016) or SIRT3-dependent (Wang et al., 2018) in mouse models.

Additionally, cADPR and NAADP, produced by CD38 from NAD^+^ catabolism, are both important Ca^2+^-mobilizing second messengers (Lee et al., 1989; Lee and Aarhus, 1995). cADPR activates ryanodine receptors and triggers the release of Ca^2+^ from the endoplasmic reticulum to the cytosol (Ogunbayo et al., 2011). NAADP is the most potent Ca^2+^-mobilizing second messenger known to date with a working concentration ranging from picomolar to low nanomolar range (Galione, 2019). The receptor for NAADP remains an active pursuit but recent studies suggested a new class of Ca^2+^-release channel, two-pore channels localized on the endolysosomes are likely to be the target for NAADP (Pitt et al., 2010; Galione, 2019). cADPR and NAADP are required for excitation-contraction coupling in the cardiomyocytes (Rakovic et al., 1996; Collins et al., 2011). Excessive production of these two messenger molecules by CD38 contributes to the pathogenesis of cardiac hypertrophy and arrhythmia (Rakovic et al., 1999; Nebel et al., 2013).

### PARylation and DNA Damage

Protein poly ADP-ribosylation (PARylation) is a reversible posttranslational protein modification in response to DNA damage (Langelier et al., 2018). PARP hydrolyzes NAD^+^ to build up the PAR chains, which are subsequently degraded by PAR glycohydrolase to terminate the PAR signal (Feng and Koh, 2013). Among 17 PARP family members, PARP1 accounts for over 90% of PARP catalytic activity (Alemasova and Lavrik, 2019). The activity of PARP1 can be activated to as much as 500-fold by DNA double strand breaks (D’amours et al., 1999). Similar to CD38, PARP1 has a K_m_ [50 μM (Ame et al., 1999), 59 μM (Mendoza-Alvarez and Alvarez-Gonzalez, 1993)] far below physiological range of NAD^+^ levels. Additionally, PARylation can be very extensive as the chain length can reach over 200 units on the target proteins (D’amours et al., 1999). Therefore, excessive activation of PARP may lead to rapid depletion of intracellular NAD^+^.

Increased PARP level has been found in human failing hearts (Pillai et al., 2005b). Reactive oxygen and nitrogen species generated from heart injuries induce DNA damage and activate PARP (Pacher and Szabo, 2007). In cardiomyocyte, PARP level has a linear correlation with the degree of cardiac hypertrophy induced by either swimming exercise or aortic banding (Pillai et al., 2005b). Overexpression of PARP in cardiomyocytes reduced the NAD^+^ and ATP contents by as much as 60% and causes more than 50% of cell death within 48 h (Pillai et al., 2005a). The PARP-induced cardiomyocyte death is mainly mediated by SIRT1 deactivation as ectopic expression or pharmacological activation of SIRT1 prevents the PARP-induced cell death (Pillai et al., 2005a). Elevation of cardiac PARP level has also been found in patients with atrial fibrillation (AF) (Zhang et al., 2019). Either NAD^+^ repletion or PARP inhibition ameliorates the contractile dysfunction in HL-1 atrial cardiomyocytes and Drosophila heart tubes after tachypacing (Zhang et al., 2019). Modulation of PARP activity by PARP inhibitors in a number of preclinical models showed improvement in the cellular energy status and cardiac function as well as attenuation in inflammation and cell death, which has been extensively reviewed elsewhere (Pacher and Szabo, 2007; Henning et al., 2018).

### Sirtuins and Deacetylation

Sirtuins or SIRTs have attracted substantial attention since their life extending capability was first discovered in yeast in 1999 (Kaeberlein et al., 1999). The subsequent discovery that SIRTs use NAD^+^ as an essential substrate led to the idea that NAD^+^ supplementation may be beneficial in prolonging life and healthy aging (Imai et al., 2000; Landry et al., 2000). It is now well established that SIRTs are NAD^+^-dependent deacetylases, which are involved in almost every aspect of human physiology including cell metabolism, cell survival, DNA repair, transcription regulation, inflammation and circadian rhythm (Kupis et al., 2016). SIRTs catalyze the protein deacetylation by transferring the acetyl group from a target protein to its co-substrate NAD^+^, which is then broken down into NAM and 2’-O-acetyl-ADPR (Sauve et al., 2001; Feldman et al., 2012). So far seven SIRT members have been discovered in mammalian cells with distinct subcellular localizations (Kupis et al., 2016). SIRT1, which is the most extensively studied SIRT, as well as SIRT6 and SIRT7, are primarily located in the nucleus. SIRT2 resides in the cytoplasm. SIRT3, SIRT4 and SIRT5 are primarily found in the mitochondria.

Sirtuin activity and NAD^+^ levels have an intricate relationship. Although SIRTs are considered NAD^+^-consuming enzymes, in most physiological situations SIRTs activation does not lead to cellular NAD^+^ depletion due to their enzymatic kinetics (Canto et al., 2015; Anderson et al., 2017; Katsyuba et al., 2020). The K_m_ of SIRT1 [94 μM (Pacholec et al., 2010), 171 μM (Smith et al., 2009), 750 μM (Madsen et al., 2016), 888 μM (Gerhart-Hines et al., 2011)], SIRT3 [280 μM (Jin et al., 2009), 880 μM (Hirschey et al., 2011)] and SIRT5 [26 μM (Roessler et al., 2015), 200 μM (Madsen et al., 2016)] lie within the physiological range of NAD^+^, thus they are unlikely to cause NAD^+^ depletion, however, their activities are highly dependent on the cellular NAD^+^ levels. Significant disturbance on NAD^+^ homeostasis can lead to abnormal SIRT1/3/5 activity which is often seen in human diseases, particularly age-related diseases such as neurodegenerative diseases, CVD and cancer (Carafa et al., 2012). In contrast, the K_m_ values of SIRT2 [83 μM (Borra et al., 2004)], SIRT4 [35 μM (Laurent et al., 2013)], SIRT6 [13 μM (Kugel et al., 2015)] for NAD^+^ are well below the physiological range of NAD^+^, so that the level of NAD^+^ will not be rate-limiting for those SIRT members, instead, activation of these low-K_m_ SIRTs may cause a rapid depletion of cellular NAD^+^ at least in theory, although supporting evidence is still lacking. Further, SIRT1 and SIRT6, directly regulate NAD^+^ biosynthesis through NAMPT (Audrito et al., 2020), the rate-limiting enzyme in the NAD^+^ salvage pathway (Figure 3). SIRT1 negatively regulates the expression of NAMPT through CLOCK:BMAL1 complex, an important mechanism that regulates the oscillatory NAD^+^ levels in a circadian fashion (Nakahata et al., 2009; Ramsey et al., 2009). In addition, deacetylation of NAMPT by SIRT1 (Yoon et al., 2015) or SIRT6 (Sociali et al., 2019) enhances NAMPT enzymatic activity and eNAMPT release.

Sirtuins play a vital role in maintaining normal cardiac function and are involved in various CVD (Sundaresan et al., 2011; Bindu et al., 2016; Hershberger et al., 2017; Ianni et al., 2018). In I/R injury model, SIRT1 (Hsu et al., 2010), SIRT3 (Porter et al., 2014), SIRT5 (Boylston et al., 2015) and SIRT6 (Wang et al., 2016) have all been shown to play a cardioprotective role. For instance, Hsu et al. (2009) demonstrated that I/R injury is associated with a reduction in NAMPT expression and depletion of NAD^+^ in heart, which leads to a SIRT1-dependent inhibition of autophagy and activation of apoptosis. SIRT3 also protects the heart from I/R injury by attenuating the myocardial oxidative stress and apoptosis through the SIRT3/forkhead box (FOX) O3/manganese superoxide dismutase (MnSOD) signaling pathway (Chang et al., 2019). Additionally, our recent study revealed that KLF15 deficiency caused a reduction in MnSOD activity due to hyperacetylation at MnSOD^K122^, resulting in elevated oxidative stress and increased susceptibility of myocardium to I/R injury in mouse heart (Li et al., 2020). The KLF15 deficiency-induced oxidative stress in cardiomyocyte can be ameliorated by NMN, which is predominantly mediated by SIRT3 (unpublished data). Because mitochondrial NAD^+^ pool is the largest subcellular NAD^+^ pool in the cardiomyocytes (see section “Subcellular Compartmentalization of NAD^+^”), our study suggests that SIRT3, the main deacetylase in mitochondria (Lombard et al., 2007; Hebert et al., 2013), may be more sensitive to the NAD^+^ level change than any other SIRT in the myocardium.

SIRT2-7 all showed a protective effect against cardiac hypertrophy and fibrosis in mouse models (Sundaresan et al., 2012; Ryu et al., 2014; Sadhukhan et al., 2016; Luo et al., 2017; Tang et al., 2017; Yu et al., 2017) whereas the effect of SIRT1 appears to be dependent on its expression level. Alcendor et al. (2007) showed that low to moderate level expression of SIRT1 (2.5–7.5 fold) attenuated age-associated cardiac hypertrophy, fibrosis and cardiac dysfunction, while a high level expression of SIRT1 (12.5 fold) led to spontaneous cardiac pathological remodeling and heart failure. It is possible that excessively high level of SIRT1 leads to NAD^+^ depletion and mitochondria dysfunction (Kawashima et al., 2011). The role of SIRTs in non-ischemic heart failure has not been extensively studied. Failing hearts from dilated cardiomyopathy (DCM) patients and mice hearts with mitochondrial complex I deficiency have increased level of mitochondrial protein acetylation (Horton et al., 2016; Lee et al., 2016), suggesting that SIRT3 may be protective against heart failure. In fact, cardiac overexpression of SIRT3 protected against angiotensin II- or doxorubicin-induced cardiac hypertrophy, dysfunction and fibrosis (Sundaresan et al., 2009; Pillai et al., 2016).

The roles of different SIRT isoforms in the context of arrhythmia remain to be further interrogated. Cardiac specific knockout of SIRT1 causes an arrhythmic phenotype resulting from Na_v_1.5 channel hyperacetylation and dysfunction (Vikram et al., 2017). Overexpression of SIRT2 reversed the repolarization defects in check point kinase BubR1 hypomorphic mice, although the exact molecular mechanism is still unclear (North et al., 2014).

Apart from the deacetylation activity, SIRTs can also use NAD^+^ as a co-substrate to catalyze protein deacylation (Sauve, 2010; Feldman et al., 2012). Each SIRT isoform has its unique substrate preference and deacylase activity (Rauh et al., 2013; Tong et al., 2017; Kumar and Lombard, 2018; Carafa et al., 2019; de Ceu Teixeira et al., 2019). For instance, SIRT5 has a strong affinity for negatively charged substrate (Rauh et al., 2013) and mediates protein desuccinylation, demalonylation, and deglutarylation (Du et al., 2011; Tan et al., 2014; Hirschey and Zhao, 2015; Kumar and Lombard, 2018). A recent study from Sadhukhan et al. (2016) revealed that succinyl-CoA is the most abundant acyl-CoA molecule in the heart. SIRT5 deletion causes an accumulation of protein lysine succinylation and leads to hypertrophic cardiomyopathy in mice. SIRT5-targeted proteins are mostly involved in metabolic pathways such as fatty acid β-oxidation, branched chain amino acid catabolism, and respiratory chain proteins (Boylston et al., 2015; Sadhukhan et al., 2016). These findings established a new paradigm of metabolic regulation by SIRT5-mediated lysine succinylation in the heart, distinct from the classic SIRT deacetylation-mediated regulatory pathways. Whether deacylation by other SIRT isoforms could also regulate the cardiac function remains to be explored in future studies.

### Subcellular Compartmentalization of NAD^+^

Different organelles have different membrane permeability to NAD^+^, and contain different NAD^+^-synthetic/consuming enzymes, which results in highly subcellular compartmentalization of NAD^+^ levels and NAD^+^-dependent cellular functions (Stein and Imai, 2012; Canto et al., 2015; Nikiforov et al., 2015; Cohen, 2020; Katsyuba et al., 2020; Ralto et al., 2020). In general, there are two major subcellular NAD^+^ pools in mammalian cells, the nucleocytoplasmic NAD^+^ pool and the mitochondrial NAD^+^ pool. The nuclear NAD^+^ pool and the cytoplasmic NAD^+^ pool are considered to be exchangeable as NAD^+^ is freely interchanged through the nuclear membrane pore (Berger et al., 2005; Houtkooper et al., 2010). By using a biosensor for NAD^+^, Cambronne et al. (2016) showed that in HEK293 cells, the cytoplasma (106 μM) and the nucleus (109 μM) had an almost identical level of free NAD^+^ under basal condition and the NAD^+^ levels decreased in a similar kinetics in response to a NAMPT inhibitor FK866. In cardiomyocytes the mitochondrial NAD^+^ pool is considerably larger than the nucleocytoplasmic pool (Stein and Imai, 2012). Over 80% of NAD^+^ is found in the mitochondrial NAD^+^ pool in rodent cardiomyocytes (Alano et al., 2007). As the inner mitochondrial membrane is impermeable to NAD^+^, the mitochondrial NAD^+^ must come from the import of NAD^+^ or NAD^+^ precursors from cytosol via transporters or indirect exchange through the malate-aspartate shuttle (MAS) where NAD^+^ is transferred out of the mitochondria in exchange for NADH into the mitochondria (Bakker et al., 2001; Nielsen et al., 2011; Satrustegui and Bak, 2015). Based on a comprehensive analysis of subcellular enzyme localizations and NAD^+^ precursors, Nikiforov et al. (2015) suggested that NMN is the mitochondria precursor for NAD^+^ generation. Another recent study using isotope labeling showed that NAD^+^ may be directly imported into mitochondria from cytosol (Davila et al., 2018).

Modulation of compartment-specific enzymes that consume or generate NAD^+^ can lead to changes in specific subcellular NAD^+^ pools. For instance, PARP inhibitor Tiq-A prevented the reduction in nuclear NAD^+^ level induced by inhibiting NAMPT, indicating that nuclear NAD^+^ level largely depends on PARP activity (Cambronne et al., 2016). Knockdown of nuclear NMNAT1 and cytoplasmic NMNAT2 lowered the level of nucleocytoplasmic NAD^+^, while knockdown of mitochondrial NMNAT3 reduced the mitochondrial NAD^+^ level (Cambronne et al., 2016).

Catabolic enzymes involved in fuel metabolism as well as SIRTs are highly compartmentalized and are regulated by local NAD^+^ levels. Changes in a specific subcellular NAD^+^ pool can alter the activity of these enzymes and related biological processes occurred in the specific subcellular compartment. The details have been recently reviewed elsewhere (Verdin, 2015; Matasic et al., 2018; Katsyuba et al., 2020).

## NAD^+^ in CVD

**FIGURE 5:**
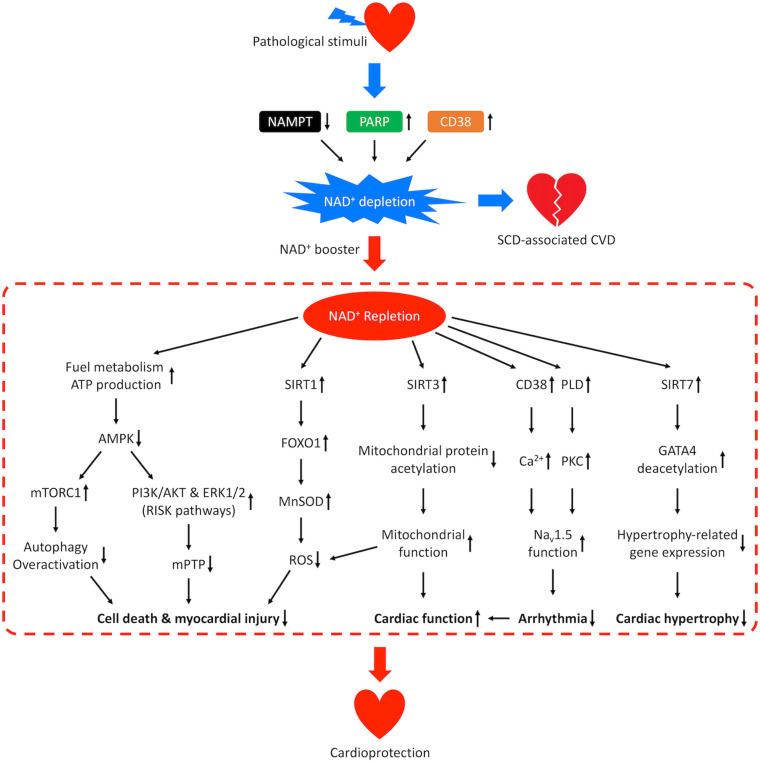
Mechanisms of cardioprotection from NAD^+^ repletion. Pathological stimuli that predispose the heart to SCD-associated CVD may result in reduced activity of NAMPT and increased activities of CD38 and PARP, which leads to NAD^+^ depletion and disease progression. Repletion of NAD^+^ by supplementation of NAD^+^ boosters confers cardioprotective effects through multiple signaling pathways. NAD^+^ repletion enhances catabolism and stimulates ATP production resulting in downregulation of AMPK, which prevents overactivation of autophagy, promotes the PI3K/AKT and ERK1/2 pro-survival kinase pathways (RISK pathways), and inhibits the mPTP-induced cell death. NAD^+^ repletion also activates SIRT1/FOXO1/MnSOD pathway that increases the clearance of ROS, SIRT3-dependent mitochondrial protein deacetylation that restores mitochondrial function and SIRT7-dependent deacetylation of GATA4 which inhibits hypertrophy-related gene expression. These mechanisms lead to reduced myocardial injury and hypertrophic remodeling as well as improved cardiac function. In addition, NAD^+^ repletion activates CD38-mediated Ca^2+^ signaling and PKC-dependent phosphorylation which improves the function of cardiac sodium channel Na_V_1.5 and lowers the risk for arrhythmia.

Both reductions in NAD^+^ biosynthesis and activation of NAD^+^-consuming enzymes can cause NAD^+^ depletion, which in turn may lead to dysregulation of numerous vital cellular functions, including fuel metabolism, SIRT-dependent regulation and CD38-mediated Ca^2+^ signaling. Chronic dysregulation of NAD^+^-dependent cell functions ultimately results in the development of CVD. An increasing number of studies, particularly in rodent models, have shown that boosting NAD^+^ is beneficial for CVD (Matasic et al., 2018; Yoshino et al., 2018; Hosseini et al., 2019b). Elevation of NAD^+^ levels can be achieved by supplementing NAD^+^, NAD^+^ precursors or modulating activities of enzymes responsible for NAD^+^ generation or degradation such as NAMPT, PARP and CD38 (Figure 5). In this review, we will focus on the *in vivo* evidence supporting the cardioprotective effects of NAD^+^ restoration in etiologies and risk factors for SCD, including I/R injury, heart failure and arrhythmia.

### I/R Injury

Ischemic heart disease is the main cause of SCD in the general population (El-Sherif et al., 2017). Ischemia causes ATP depletion and cardiomyocyte necrosis. Reperfusion restores the energy supply, however, paradoxically imposes oxidative stress by ROS production (Kalogeris et al., 2016). The NAD^+^ levels are reduced in the myocardium after both ischemia and I/R injury (Yamamoto et al., 2014). A variety of NAD^+^ precursors including NA (Trueblood et al., 2000), NAM (Sukhodub et al., 2010), NMN (Hosseini et al., 2019a), and NAD^+^ itself (Zhang et al., 2016; Zhai et al., 2019), have been consistently shown to restore the NAD^+^ levels, reduce myocardial infraction (MI) size and protect against I/R injury dysfunction *in vivo*.

Activation of NAD^+^-biosynthetic enzymes or inhibition of NAD^+^-consuming enzymes have also shown cardioprotective effects in response to I/R. Hsu et al. (2009) showed that cardiac expression of NAMPT was reduced by I/R in mice. Cardiac-specific overexpression of NAMPT increased the NAD^+^ levels and reduced MI size and cardiomyocyte apoptosis in response to ischemia and I/R (Hsu et al., 2009). On the other hand, the activity of CD38 is elevated by more than 5-fold in the post-ischemic heart (Reyes et al., 2015). CD38 deficiency reduced the MI size in response to I/R injury (Guan et al., 2016). Inhibition of PARP by genetic deletion or pharmacological inhibitors have also shown therapeutic effects against I/R injury in various animal models, which has been extensively reviewed elsewhere (Zingarelli et al., 1998, 2003, 2004; Grupp et al., 1999; Pieper et al., 2000; Yang et al., 2000; Zhou et al., 2006; Pacher and Szabo, 2007).

Multiple signaling pathways are involved in mediating the cardioprotective effects from NAD^+^ restoration. First, the SIRT1/FOXO1 pathway is upregulated by NAD^+^ restoration in response to I/R injury (Yamamoto et al., 2014; Guan et al., 2016; Zhang et al., 2016). Elevated NAD^+^ levels lead to SIRT1 activation and deacetylation of cytosolic FOXO1, which promotes its nuclear translocation (Frescas et al., 2005; Hsu et al., 2010). Nuclear FOXO1 then activates transcription of antioxidants such as *MnSOD*, which leads to increased ROS clearance and ameliorates the oxidative stress in the heart (Brunet et al., 2004; Hsu et al., 2010). FOXO1 also activates autophagic flux by transcriptional activation of *Rab7*, which preserves energy during ischemia (Hariharan et al., 2010). Second, the PI3K/AKT and ERK1/2 pro-survival kinase pathways, or the so-called reperfusion injury salvage kinase (RISK) pathways (Rossello and Yellon, 2018), are upregulated by NAD^+^ restoration under I/R conditions (Lim et al., 2008; Sukhodub et al., 2010). NAD^+^ restoration activates RISK pathways possibly through downregulation of AMPK activity. Elevation of NAD^+^ level increases glycolysis and fatty acid β-oxidation which generates more ATP and decreases the AMP/ATP ratio, which reduces AMPK activity (Dyck and Lopaschuk, 2006). AMPK has been shown to suppress both PI3K/AKT signaling pathway (Tzatsos and Tsichlis, 2007) and ERK1/2 signaling pathway (Meng et al., 2011), therefore inhibition of AMPK activates the RISK pathways. RISK pathways are rapidly activated during the first few minutes of reperfusion and have been proposed as a potential therapeutic target for cardioprotective intervention (Hausenloy and Yellon, 2004; Rossello and Yellon, 2018). Noma et al. demonstrated that activation of RISK pathways through activation of EGFR signaling confers cardioprotective effect *in vivo* (Noma et al., 2007; Pinilla-Vera et al., 2019). Mechanistically, RISK pathways reduce the opening probability of mitochondrial permeability transition pore (mPTP) and mPTP-induced cell death *in vitro* (Davidson et al., 2006). The activation of RISK pathways also upregulates the activity of a cardiac ATP-sensitive K^+^ channel and leads to reduced Ca^2+^ entry, muscle contractility and energy consumption (Sukhodub et al., 2010), which potentially protects the myocardium during ischemia (Nichols, 2016).

In summary, repletion of NAD^+^ levels by either direct supplementation of NAD^+^ precursors or targeting the NAD^+^-biosynthetic/consuming enzymes activity may be a potential therapeutic strategy to mitigate the cardiac injury from I/R. The SIRT1/FOXO1 and RISK pathways are two major players that mediate the beneficial effects through reducing ROS level, inhibiting cell death and preserving energy.

### Heart Failure and Pathological Remodeling

Human failing hearts from patients with DCM showed reductions in NAD^+^ level and NAD^+^/NADH ratio (Horton et al., 2016; Lee et al., 2016). The cardioprotective effects of NAD^+^ and its precursors (NR, NMN, and NAM) have been demonstrated in a number of small animal models for heart failure (Riehle and Bauersachs, 2019), including (1) genetic models in which the mitochondrial function is disrupted such as Frataxin (*Fxn*)-knockout model (Puccio et al., 2001; Wagner et al., 2012) [NMN (Martin et al., 2017)], NADH dehydrogenase [ubiquinone] iron-sulfur protein 4 (*Ndufs4*)-knockout model (Karamanlidis et al., 2013) [NMN (Lee et al., 2016)] and transferrin receptor (*Tfrc*)-knockout model [NR (Xu et al., 2015)]; (2) genetic models in which a cytoskeletal component is dysfunctional such as MDX mice model (Quinlan et al., 2004) [NR (Ryu et al., 2016)]; (3) genetic models in which a cardiac transcription factor is deficient such as the serum response factor (*Srf*)-knockout model (Diguet et al., 2011) [NR (Diguet et al., 2018)]; (4) non-genetic models, including pressure overload (Rockman et al., 1994) [NR (Diguet et al., 2018) and NMN (Lee et al., 2016)], volume overload (Liu et al., 1991) [NAM (Cox et al., 2002)], angiotensin II-induced hypertension (Wollert and Drexler, 1999) [NAD^+^ (Pillai et al., 2010)], and isoproterenol-induced (Oudit et al., 2003) [NAD^+^ (Pillai et al., 2010)] heart failure models. The cardioprotective effects of boosting NAD^+^ level observed in various animal models suggest that raising the NAD^+^ levels may be a promising therapeutic strategy for heart failure (Walker and Tian, 2018).

Mitochondrial dysfunction and mitochondrial protein hyperacetylation are causally linked to the development of heart failure (Rosca and Hoppel, 2013; Lee and Tian, 2015; Horton et al., 2016; Zhou and Tian, 2018). NAD^+^ repletion restored SIRT3 activity and reversed mitochondrial protein hyperacetylation in the failing hearts and improved cardiac function (Lee et al., 2016). Using acetylome analysis, Lee et al. (2016) identified a subgroup of NAD^+^/NADH-sensitive mitochondrial proteins, the hyperacetylation of which are highly associated with the development of heart failure, including MAS components and mPTP regulatory proteins. Apart from SIRT3, expression of SIRT1 was found to be reduced in patients with advanced heart failure (Lu et al., 2014). Similar to that in I/R injury, SIRT1/FOXO1 pathway was suppressed, which reduced the expression of antioxidants such as MnSOD and Thioredoxin1, whereas the expression of pro-apoptotic molecule BAX was increased (Lu et al., 2014). Therefore, NAD^+^ repletion may reduce oxidative stress and apoptosis in failing hearts through activation of SIRT1/FOXO1 pathway. In addition, a recent study showed that NAD^+^ repletion by NMN supplement activates SIRT7/GATA binding protein 4 (GATA4) pathway which confers anti-hypertrophic effects in response to pressure overload (Yamamura et al., 2020). SIRT7 directly interacts and deacetylates GATA4, which regulates its transcriptional activity and suppresses the cardiac hypertrophy-related gene expression (Yamamura et al., 2020).

The cardioprotective effects of NAD^+^ repletion in heart failure may also come from inhibition of AMPK signaling pathways. AMPK is activated as a result of energy depletion and metabolic remodeling in the failing heart (Azevedo et al., 2013; Doenst et al., 2013). NAD^+^ repletion can suppress the elevated AMPK activity in heart failure (Lee et al., 2016; Ryu et al., 2016; Diguet et al., 2018). As mentioned above, NAD^+^ may inhibit AMPK activity indirectly through the activation of glycolysis and fatty acid β-oxidation. The effects of AMPK activation on cardiac function depends on the stage and the severity of the heart failure. In the early stage before decompensation, activation of AMPK is considered to be an adaptive mechanism which may help maintain the physiological level of autophagy (Nakamura and Sadoshima, 2018) by inhibiting mTORC1 signaling (Kim et al., 2011) and delay the transition from cardiac remodeling to heart failure (Pillai et al., 2010; Beauloye et al., 2011). Once decompensation occurs, chronic activation of AMPK can actually be detrimental to cardiac function. Overactivation of AMPK may lead to uncontrolled autophagy which triggers autophagy-dependent cell death and myocardial injury (Zhu et al., 2007; Sciarretta et al., 2018; Kaludercic et al., 2020). Further, long-term activation of AMPK suppresses the pro-survival RISK signaling pathways (Tzatsos and Tsichlis, 2007; Meng et al., 2011). Therefore, NAD^+^ repletion-induced inhibition on AMPK signaling pathway may be particularly beneficial for late stage heart failure.

Intriguingly, modulation of NAMPT activity has shown contradictory effects on heart failure. Lee et al. (2016) showed that overexpression of cardiac NAMPT partially restored the cardiac function after pressure overload and completely reversed the isoproterenol-induced cardiac dysfunction in the mitochondrial complex I defective mouse model. However, Byun et al. (2019) showed that both gain and loss of NAMPT function exacerbated the pressure overload-induced heart failure. On the other hand, animal studies on PARP inhibition consistently showed significant preservation in cardiac function and promotion in survival rate in heart failure models (Booz, 2007; Pacher and Szabo, 2007; Halmosi et al., 2016; Henning et al., 2018). In addition, knockout of CD38 was shown to be cardioprotective against angiotensin II−induced cardiac hypertrophy in mouse (Guan et al., 2017). This discrepancy may be due to the proinflammatory effect of eNAMPT (Montecucco et al., 2013; Byun et al., 2019). Further, overexpression of NAMPT may not necessarily elevate NAD^+^ level if the amount of NAM is limited (Byun et al., 2019). NAMPT overexpression may also lead to excessive amount of NAD^+^ and overactivation of SIRT1 which has been shown to impair mitochondrial and cardiac function (Kawashima et al., 2011).

In summary, NAD^+^ repletion could provide cardioprotective effects against cardiac remodeling and heart failure through multiple mechanisms, including reduction of oxidative stress, suppression of hypertrophy-related gene expression, prevention of autophagy overactivation, restoration of mitochondrial function and activation of RISK pro-survival pathways. These findings indicate that NAD^+^-boosting intervention may be a potential therapeutic strategy for cardiac remodeling and heart failure.

### Arrhythmia

We have only started to explore the effects of NAD^+^ on arrhythmia. Cardiac sodium current (I_Na_) upstroke during depolarization phase is predominantly mediated by Na_v_1.5 channel, which is the major voltage-gated sodium channel in the heart (Veerman et al., 2015). Mutations in Na_v_1.5 are associated with a number of cardiac diseases such as long QT syndrome, AF and cardiomyopathy (Song and Shou, 2012; Shy et al., 2013; Han et al., 2018; Wilde and Amin, 2018). Matasic et al. (2020) showed that NR increased I_Na_ and reduced residual late I_Na_ *in vitro*, and more importantly administration of NR decreased QTc *in vivo*. Mechanistically, NR supplementation elevates NADH level which increases phospholipase D (PLD) activity leading to activation of PKC signaling pathway (Martin et al., 2017). PKC phosphorylation at S1503 in Na_v_1.5 channel (Matasic et al., 2020) modulates the channel conductance and improves the I_Na_ profile in the heart (Valdivia et al., 2009; Martin et al., 2017). The anti-arrhythmic effect of NAD^+^ supplementation may not be exclusively dependent on the NAD^+^ levels. Liu et al. (2013) demonstrated that administration of NAD^+^ restored I_Na_ from 60% to 97% in mouse with non-ischemic cardiomyopathy and improved conduction velocity in human myopathic hearts. However, the I_Na_ restoration by NAD^+^ was diminished by CD38 inhibitor pelargonidin, suggesting that CD38-mediated Ca^2+^ signaling rather than the intracellular NAD^+^ level plays a direct role (Liu et al., 2013).

In patients with AF and tachypaced cardiomyocytes, PARP1 was found to be hyperactive, which leads to NAD^+^ depletion (Zhang et al., 2019). Excessive production of ROS observed in AF (Youn et al., 2013; Xie et al., 2015; Liang et al., 2018) may be associated with high level of DNA damage, which leads to activation of PARP1. Inhibition of PARP1 restored the NAD^+^ content, reduced oxidative stress and improved cardiomyocyte contractility in rat atrial cardiomyocytes and Drosophila hearts with tachypacing (Zhang et al., 2019).

Apart from Na_v_1.5, future study should look into other NAD^+^-regulated ion transporters (Kilfoil et al., 2013) that are important for cardiac electrophysiology and their potential implications in anti-arrhythmic effects, such as Kv4.2 (Tur et al., 2016, 2017). In addition, severe NAD^+^ depletion (>95%) sabotages the ability to regenerate ATP (Del Nagro et al., 2014) which may potentially impair the normal phosphorylation state and function of ion transporters in the heart (Ismailov and Benos, 1995; Grant, 2009; Bartos et al., 2015). Future study may explore whether NAD^+^ repletion can restore a global level of phosphorylation on cardiac ion transporters after severe NAD^+^ depletion.

In summary, there is an emerging evidence suggesting that NAD^+^ replenishment plays important roles in cardiac arrhythmia.

## The Emerging NAD^+^-Boosting Therapy

There has been a rapidly growing attention and expectation on the clinical usage of NAD^+^-boosting therapy for diseases associated with NAD^+^ depletion and metabolic syndrome. Over 300 clinical trials (clinicaltrials.gov) have been conducted to test the therapeutic potential of NAD^+^ precursors on Alzheimer’s disease, psoriasis, obesity, diabetes, chronic kidney disease, dyslipidemia, and CVD etc (Katsyuba et al., 2020). Currently, NA (Niaspan) is the only US Food and Drug Administration (FDA)-approved NAD^+^ precursor product and is used to treat dyslipidemia in the US (Villines et al., 2012; Rajman et al., 2018). However, NR, NA, NMN, and NAM are all natural products and their use does not require FDA approval. As a result, they are taken as food supplements for a broad spectrum of indications in many situations but without evaluation from well-controlled clinical studies.

### Human Studies on SCD-Associated CVD

As a basis for clinical translation, the safety of NAD^+^-boosting interventions in high-risk groups for CVD has been well evaluated (Dellinger et al., 2017; Martens et al., 2018). A recently published review from Katsyuba et al. (2020) summarized the human studies with NAD^+^ boosters in different disease settings. Here, we will focus on human studies on SCD-associated CVD.

Since the lipid-lowering effect of NA was found in 1950s (Altschul et al., 1955; Parsons et al., 1956), most of the human studies on coronary artery disease (CAD) or ischemic heart disease have chosen NA as the NAD^+^ booster of choice. Initiated in 1962, the Coronary Drug Project demonstrated that subjects treated with NA had a 10% reduction of the serum cholesterol levels (Berge and Canner, 1991). Moreover, NA was shown to reduce the levels of low-density lipoprotein while increase the level of high-density lipoprotein, and the combination therapy with colestipol (Brown et al., 1990) or statins (Guyton et al., 2008; Sang et al., 2009) offered even greater lipid lowering benefits. Consistent with the improved lipid profile, NA offers additional preventive effects on atherosclerotic progression in patients receiving colestipol (Blankenhorn et al., 1987a, b; Brown et al., 1990) or statins (Brown et al., 2001; Taylor et al., 2004; Villines et al., 2010; Boden et al., 2011; Landray et al., 2014). Further, NA-treated subjects had a lower incidence of definite, non-fatal MI (10% vs. 14% in placebo group) (Berge and Canner, 1991). Most importantly, the NA treated group, although showing a trend toward lowering mortality at the conclusion of the trial, actually showed a significant (11%, *Z* = −3.52, *p* < 0.0004) reduction of mortality in the extended follow-up study 9 years from the conclusion of the active study. Subjects taking NA previously showed a 1.6 year extension of life (Berge and Canner, 1991). However, another two clinical trials with patients receiving statin-based therapy showed that the addition of NA failed to provide clinical benefits in reducing the risk of major vascular events or composite cardiovascular death (Boden et al., 2011; Landray et al., 2014). Because the NAD^+^ level is oscillating in a circadian fashion in heart, one possible explanation for the inconsistent results is that the timing of implementing NAD^+^-boosting intervention may have a significant impact on the clinical outcome. As a supportive evidence for this notion, our recent study showed that NMN administration elevated NAD^+^ level and reduced MI size in mice after I/R injury at ZT2 when the NAD^+^ level was at nadir, however, the improvement was not observed when NMN was supplemented at ZT14 when the NAD^+^ level was at peak (Li et al., 2020). Therefore, an optimal time window may be critical for NAD^+^-boosting therapy, which needs to be carefully examined in future studies. Another explanation might be the lack of a pharmacological effective dose. In fact, most of the human studies reported so far with NAD^+^ boosters, including those on non-CVD, did not provide evidence for elevated NAD^+^ level in experimental subjects (Katsyuba et al., 2020).

For heart failure, two small-scale pilot clinical studies, one with 5 and the other with 30 patients, aiming to assess the safety and feasibility of NR have been completed but the results have not been published yet (clinicaltrials.gov). Another pilot study on NAM is in the progress of recruiting 60 heart failure patients (clinicaltrials.gov). To date, no clinical trial has been conducted to examine the role of NAD^+^-boosting strategies on arrhythmia (clinicaltrials.gov). The effects of NAD^+^ boosters on patients with heart failure and arrhythmia remain to be investigated in future studies.

In summary, current human studies have shown that NAD^+^-boosting therapy can reduce mortality (Carlson and Rosenhamer, 1988; Berge and Canner, 1991; Brown et al., 2001; Landray et al., 2014) and provide moderate clinical benefits for patients with CAD. However, conflicting results on critical clinical outcomes such as incidence of composite mortality and major vascular events have raised the concern that whether NAD^+^-boosting therapy can ultimately become a primary treatment for CAD and other CVD. Several important aspects may help overcome these hurdles. First, it is critical to determine the effective dose of NAD^+^ boosters for each individual patient. Direct measurement for NAD^+^ level or NAD^+^ metabolome from accessible samples [i.e., plasma (Grant et al., 2019)] should be considered. It is possible to achieve the effective therapeutic level, novel NAD^+^ precursors or novel pharmaceutical formulations are required. Second, the optimal time window for NAD^+^ booster supplementation remains to be established in human subjects. NAD^+^-boosting therapy should coordinate with the intrinsic circadian oscillation of NAD^+^ level in human body so that maximal beneficial effects can be achieved. With a more nuanced understanding of NAD^+^ biology in the heart and clinical studies designed with more sophistication, we anticipate that NAD^+^-boosting therapy would ultimately harness its potential for SCD-associated CVD.

## Author Contributions

LZ conceived the manuscript. WX, LL, and LZ wrote the manuscript. All authors contributed to the article and approved the submitted version.

## Conflict of Interest

The authors declare that the research was conducted in the absence of any commercial or financial relationships that could be construed as a potential conflict of interest.

## Abbreviations

ADPR: ADP-ribose
AF: atrial fibrillation
BMAL1: brain and muscle Arnt-like protein 1
CAD: coronary artery disease
cADPR: cyclic ADPR
CLOCK: circadian locomoter output cycles protein kaput
CVD: cardiovascular diseases
DCM: dilated cardiomyopathy
eNAMPT: extracellular NAMPT
ENT: equilibrative nucleoside transporter
ETC: electron transport chain
FOX: forkhead box
GATA4: GATA binding protein 4
I/R injury: ischemia/reperfusion injury
I_Na_: sodium current
KLF15: Kruppel-like factor 15
K_m_: Michaelis constant
MAS: malate-aspartate shuttle
MI: myocardial infarction
mPTP: mitochondrial permeability transition pore
NA: nicotinic acid
NAADP: nicotinic acid adenine dinucleotide phosphate
NAD^+^: nicotinamide adenine dinucleotide
NADP^+^: nicotinamide adenine dinucleotide phosphate
NAM: nicotinamide
NAMPT: NAM phosphoribosyltransferase
NMN: nicotinamide mononucleotide
NMNAT1-3: NMN adenyltransferase 1-3
NOXs: NADPH oxidases
NR: nicotinamide riboside
NRK1/2: NR kinase 1/2
PAR: poly ADP-ribose
PARP: PAR polymerases
PARylation: poly ADP-ribosylation
PLD: phospholipase D
PPP: pentose phosphate pathway
RISK: reperfusion injury salvage kinase (PI3K/AKT and ERK1/2)
ROS: reactive oxygen species
SCD: sudden cardiac death
SIRT: sirtuin
SLC12A8: solute carrier family 12 member 8
TCA: tricarboxylic acid
Trp: tryptophan.
